# Intervention to Lower Household Wood Smoke Exposure in Guatemala Reduces ST-Segment Depression on Electrocardiograms

**DOI:** 10.1289/ehp.1002834

**Published:** 2011-06-13

**Authors:** John McCracken, Kirk R. Smith, Peter Stone, Anaité Díaz, Byron Arana, Joel Schwartz

**Affiliations:** 1 Department of Environmental Health, Harvard School of Public Health, Boston, Massachusetts, USA; 2 Environmental Sciences Division, University of California, Berkeley, California, USA; 3 Brigham and Women’s Hospital, Boston, Massachusetts, USA; 4 Center for Health Studies, Universidad del Valle, Guatemala City, Guatemala

**Keywords:** biomass fuel, cardiovascular disease, electrocardiography, indoor air pollution, RESPIRE

## Abstract

Background: A large body of evidence suggests that fine particulate matter (PM) air pollution is a cause of cardiovascular disease, but little is known in particular about the cardiovascular effects of indoor air pollution from household use of solid fuels in developing countries. RESPIRE (Randomized Exposure Study of Pollution Indoors and Respiratory Effects) was a randomized trial of a chimney woodstove that reduces wood smoke exposure.

Objectives: We tested the hypotheses that the stove intervention, compared with open fire use, would reduce ST-segment depression and increase heart rate variability (HRV).

Methods: We used two complementary study designs: *a*) between-groups comparisons based on randomized stove assignment, and *b*) before-and-after comparisons within control subjects who used open fires during the trial and received chimney stoves after the trial. Electrocardiogram sessions that lasted 20 hr were repeated up to three times among 49 intervention and 70 control women 38–84 years of age, and 55 control subjects were also assessed after receiving stoves. HRV and ST-segment values were assessed for each 30-min period. ST-segment depression was defined as an average value below –1.00 mm. Personal fine PM [aerodynamic diameter ≤ 2.5 μm (PM_2.5_)] exposures were measured for 24 hr before each electrocardiogram.

Results: PM_2.5_ exposure means were 266 and 102 μg/m^3^ during the trial period in the control and intervention groups, respectively. During the trial, the stove intervention was associated with an odds ratio of 0.26 (95% confidence interval, 0.08–0.90) for ST-segment depression. We found similar associations with the before-and-after comparison. The intervention was not significantly associated with HRV.

Conclusions: The stove intervention was associated with reduced occurrence of nonspecific ST-segment depression, suggesting that household wood smoke exposures affect ventricular repolarization and potentially cardiovascular health.

Approximately 3 billion people depend on biomass (e.g., wood, crop residues, animal dung) and coal for household cooking and heating (Smith et al. 2004). Most of this solid fuel use occurs in developing countries, where poor households generally use open wood fires or inadequately vented stoves (Rehfuess et al. 2006). These fuel-stove combinations result in high indoor levels of fine combustion-generated particles and other pollutants, and epidemiological evidence suggests a substantial global burden of disease from respiratory health effects (Smith et al. 2004). Numerous epidemiological investigations have concluded that ambient combustion particles and secondhand tobacco smoke (SHS) are associated with cardiovascular morbidity and mortality (Brook et al. 2003, 2004; Dockery et al. 1993; He et al. 1999; Pope et al. 1995; Schwartz 1993, 1994; Thun et al. 1999), but to our knowledge no such studies have been published on the effects of indoor air pollution from woodstoves on cardiovascular disease. Notwithstanding potentially important differences between these pollution mixtures, this evidence on ambient particles and SHS, combined with the wood smoke’s toxicological properties, particularly the promotion of inflammation, blood coagulation, and oxidative stress (Naeher et al. 2007), raises concern about potential cardiovascular effects of household wood smoke. Fortunately, simple, inexpensive household-energy interventions can dramatically reduce indoor pollution (Albalak et al. 2001; Bruce et al. 2004; Ezzati and Kammen 2002; Naeher et al. 2000; Smith et al. 1993), providing unique opportunities to understand and prevent the health effects of chronic exposures to these pollutants. A recent risk assessment analysis estimated that 560,000 cardiovascular deaths could be prevented by introducing 150 million low-emission stoves in India over the next decade (Wilkinson et al. 2009), but this estimate relies on the assumption that fine particles [aerodynamic diameter ≤ 2.5 μm (PM_2.5_)] in ambient air and those from using biomass stoves have similar effects on ischemic heart disease, which is supported by little direct epidemiological evidence.

The RESPIRE (Randomized Exposure Study of Pollution Indoors and Respiratory Effects) project, the first randomized controlled trial to investigate the health effects of reducing air pollution exposures over a period of many months in a general population, installed improved chimney woodstoves in households using open wood fires for cooking in highland villages (elevation, 2,200–3,000 m) in rural Guatemala (Smith et al. 2006). The intervention stove has been shown to reduce kitchen concentrations of PM_2.5_ and carbon monoxide (Albalak et al. 2001; Bruce et al. 2004; McCracken et al. 2009; Smith et al. 1993, 2010). The trial focused on acute lower respiratory infections in infants but also created an opportunity to study the potential cardiovascular health benefits of reduced air pollution exposures. Given the limited size of the study population and duration of the trial period, we focused on subclinical measures of physiological change relevant to cardiovascular risk. Evidence that this intervention reduces blood pressure was reported previously (McCracken et al. 2007). Because combustion particles from other sources have been associated with occurrence of ST-segment depression and decreased heart rate variability (HRV) (Chahine et al. 2007; Chuang et al. 2007, 2008; Gold et al. 2005; Pope et al. 1999, 2001; Schwartz et al. 2005), we also measured these electrocardiogram outcomes. The objective of the present study was to evaluate whether the stove intervention influences the occurrence of ST-segment depression or levels of HRV during everyday activities.

## Materials and Methods

*Study population*. The study area for the RESPIRE project comprised 23 rural villages spread over an area of about 94 km^2^ in the towns of San Lorenzo and Comitancillo, San Marcos, in the western highland region of Guatemala, home to a primarily agricultural indigenous population living at an elevation of 2,200–3,000 m above sea level. A baseline survey of these villages in 2002 showed that nearly all households used solid biomass fuels (primarily wood and crop residues) for cooking, and most burned these fuels indoors with open fires. Eligibility criteria for the RESPIRE project included exclusive use of open fires for cooking. Households (*n* = 534) were recruited and randomized into control and intervention groups from October 2002 through May 2003 (Smith et al. 2006). The RESPIRE trial protocols were approved by the human subjects committees at each participating center, and all participants gave informed consent before data collection.

**Figure 1:**
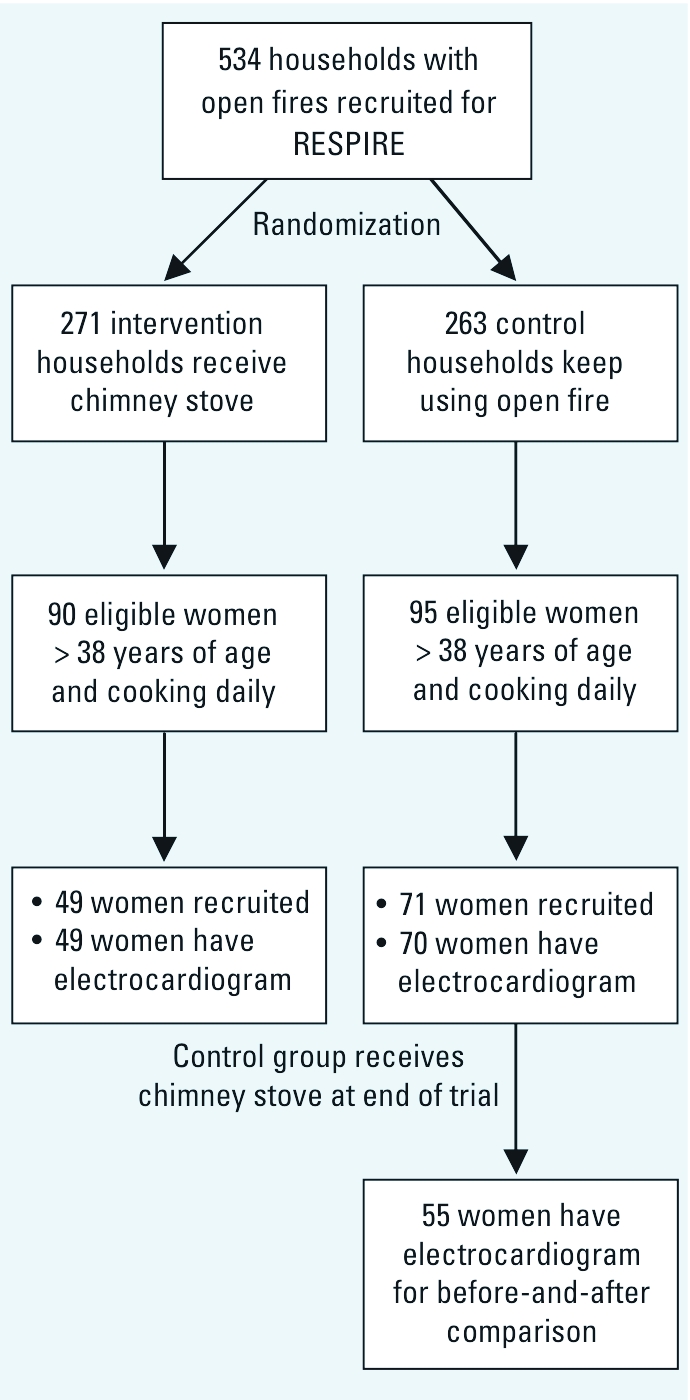
Flow chart of recruitment into the cardiovascular substudy within RESPIRE trial.

From 8 July 2003 to 4 November 2004, after randomization to the intervention (chimney stove) and control (open fire) groups, we recruited for the cardiovascular substudy women who cooked daily, were > 38 years of age, lived in one of 19 villages closest to the study headquarters, and were generally the grandmothers of children in the acute lower respiratory infection study (Figure 1). Response rates were 75% and 54% among control and intervention households, respectively, resulting in 119 participants. The control and intervention groups were similar on all measured baseline characteristics, and their demographic and socioeconomic characteristics have been described previously (McCracken et al. 2007).

At the end of the trial, the control households received the chimney stove, which allowed a before-and-after comparison.

*Measurements.* Baseline subject characteristics, such as age, ownership of assets (radio, television, bicycle, etc.), exposure to SHS, and use of a wood-fired sauna for bathing, were attained before randomization.

We measured personal suspended PM_2.5_ during the 24 hr before each Holter session by asking the participants to wear air samplers that included an Apex pump with flow set to 1.5 L/min (Casella Inc., Bedford, UK), a Triplex Sharp-Cut Cyclone for particle size selection (BGI Inc., Waltham, MA, USA) with inlet clipped above waste height, and a 37-mm Teflon filter inside a metal filter holder (McCracken et al. 2007). Then participants were outfitted with a Performer model 9000 Holter monitor (Applied Cardiac Systems, Laguna Hills, CA, USA) and monitored over 20-hr periods. The protocol was repeated on two or three occasions for most participants. Repeated measures within subjects were taken at least 3 weeks apart, and each subject’s set of two or three sessions was completed over a period of 184 days on average (range, 21–420 days). To avoid confounding by study period, on each measurement day during the trial period we measured one intervention subject and one control subject, which for convenience were chosen from the participating households that were nearest to each other.

We scanned Holter recordings from the modified V5 lead and calculated the ST-segment value 60 msec after the J-point of each beat and its deviation relative to the P-R isoelectric (resting baseline) value. We defined an ischemic episode by transient ST-segment depression > 1.0 mm (0.1 mV) compared with the resting baseline (i.e., 0 mV), lasting ≥ 1 min, separated by ≥ 5 min from other episodes, and with horizontal or downward-sloping ST-segment morphology. We also calculated 30-min average ST-segment values as a measure of nonspecific changes in ventricular repolarization, and we created a dichotomous variable for whether this value was below –1.0 mm (–0.1 mV), regardless of the slope of the ST-segment.

HRV, also calculated over 30-min intervals, was measured as standard deviation of normal-to-normal intervals (SDNN) and low-frequency (LF; 0.04–0.15 Hz) and high-frequency (HF; 0.15–0.4 Hz) components of variability. Average heart rate was also recorded for each 30-min interval.

A questionnaire conducted before and after each electrocardiogram monitoring session was used to collect information on recent exposures, such as use of a wood-fired sauna. Daily average temperature, relative humidity, and rainfall were collected by a weather station near the center of the study area.

*Statistical analyses.* To estimate the geometric mean and 95% confidence intervals (CIs) of personal PM_2.5_ exposure by study group and period, we log-transformed the PM_2.5_ measures and used mixed models with random intercepts for subjects to provide more accurate CIs that account for correlation among repeated measures.

We fitted logistic regression models for the occurrence of 30-min average ST-segment depression and linear regression models for the natural-log–transformed measures of HRV. To account for correlation among repeated measures, we assessed the intervention effects using generalized estimating equations in SAS PROC GENMOD (version 9.1; SAS Institute Inc., Cary, NC, USA), selecting autoregressive covariance within subjects and reporting sandwich estimators of variance. Statistical significance was defined as *p* < 0.05.

The between-groups study design compares the control and intervention groups during the trial period. Although randomization is expected to produce two comparable groups, because of the small sample size and postintervention selection, we adjusted for potential between-subjects confounders selected *a priori*, namely, age, body mass index (BMI), ever smoking, SHS, ownership of a wood-fired sauna, recent use of wood-fired sauna, an asset index as a measure of socioeconomic status, and time of day. The asset index is the sum of binary indicators for having a bicycle, a radio, and a television.

The before-and-after study design estimates the within-subject effect of the intervention among control subjects by comparing ECG data collected before and after adoption of the chimney stoves. People serve as their own controls, so these estimates can only be biased by factors that vary over time, and we adjusted for several time-varying covariates, including age, day of week, season (wet/dry), daily average temperature and relative humidity, daily rainfall, interactions of weather variables with season, recent use of wood-fired sauna, and time of day.

Sensitivity analyses for ST-segment depression included adjusting for average heart rate during the 30-min intervals and modeling the counts of 30-min intervals with ST values ≤ –1.0 mm per Holter session as a Poisson variable using generalized linear mixed models (SAS PROC GLIMMIX). To assess potential bias due to varying numbers of observations per subject, we repeated the between-groups and before-and-after analyses for ST-segment and HRV using only each subject’s last Holter session during the corresponding study period. We also evaluated all associations with the stove intervention within age groups < 52 and ≥ 52 years and within BMI groups < 25 and ≥ 25 kg/m^3^ and tested for interaction between these age and BMI groups and the stove intervention. Because the difference in mean personal PM_2.5_ exposure between the groups appeared to be less during the first 3 months of the trial compared with the rest of the trial, we tested for an interaction between the stove intervention and study period (first 3 months vs. the rest of the trial period). All interactions were tested by adding a term for the product of the two variables to the models.

## Results

Table 1 summarizes baseline characteristics of participants in the control and intervention groups. The median age was 50 years, and the median BMI was 24 kg/m^3^; these two important predictors of ST-segment depression were similar, on average, in the two groups. The women in this study lived at an elevation of around 2,600 m above sea level, on average. Most women in both groups had none or only one of the assets included in the index used as a surrogate for socioeconomic status (bicycle, radio, television), and the proportion of women from households with access to electricity was around 70% in both groups. Percentages of women who reported ever smoking or other sources of household air pollution, such as wood-fired saunas, kerosene lamps, and SHS exposure, were also similar between the two study groups.

**Table 1:** Baseline characteristics of women in the RESPIRE electrocardiogram study, by randomized group.

Characteristic	Control open fire (*n* = 70)	Intervention chimney stove (*n* = 49)	*p*-Value*a*
Subjects						
Age (years)		52.6 ± 11.1		54.1 ± 11.1		0.48
BMI (kg/m^2^)		24.3 ± 3.0		24.8 ± 3.2		0.39
Ever smoked (%)		11		10		0.85
Household						
Elevation (m)		2,617 ± 189		2,649 ± 183		0.35
Kitchen volume (m^3^)		47.2 ± 22.9		43.6 ± 22.8		0.42
Electricity in house (%)		73		69		0.65
Asset index*b* (%)						
0		6		8		0.50
1		55		45		
2		32		33		
3		7		14		
Other air pollution source (%)						
Wood-fired sauna		93		94		0.84
Kerosene lamp		21		14		0.34
SHS		25		18		0.37
Means ± SDs are presented for age, BMI, elevation, and kitchen volume. **a**Two-sided *p*-values from *t*-tests for continuous variables and chi-square tests for categorical variables. **b**Sum of binary indicators for having a radio, television, and bicycle in household.						

**Figure 2:**
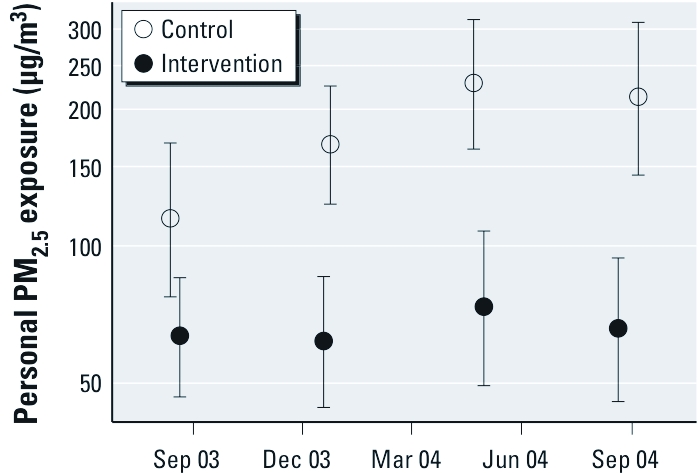
Personal 24-hr PM_2.5_ exposure (geometric means and 95% CIs) during each quartile of time during the trial period for intervention and control groups.

We maintained a substantial exposure contrast throughout the trial, although the contrast may have been slightly less during the first few months (Figure 2). The average personal PM_2.5_ exposure in the control group was 266 μg/m^3^ (minimum, 16 μg/m^3^; 25th percentile, 109 μg/m^3^; median, 201 μg/m^3^; 75th percentile, 327 μg/m^3^; maximum, 2,088 μg/m^3^), and the average in the intervention group was 102 μg/m^3^ (minimum, 3 μg/m^3^; 25th percentile, 37 μg/m^3^; median, 69 μg/m^3^; 75th percentile, 118 μg/m^3^; maximum, 832 μg/m^3^). The mixed-model–estimated geometric means of personal PM_2.5_ during the trial period were 179 and 65 μg/m^3^ for control and intervention groups, respectively, equivalent to a 64% reduction (95% CI: –73%, –53%) associated with the chimney-stove intervention compared with the open-fire group. In the before-and-after comparison, the estimated geometric means were 187 and 112 μg/m^3^ (arithmetic means, 273 and 174 μg/m^3^) before and after the stove intervention, respectively, equivalent to a somewhat smaller 40% (95% CI: –56%, –19%) reduction in personal PM_2.5_ after introduction of the chimney stove in control households. The intervention group had been cooking with the chimney stove for 293 days on average (range, 2–700 days) when we took measures during the trial period, whereas control households had been using the improved stoves for 63 days (range, 0–342 days) on measurement occasions after the trial period.

Table 2 shows the numbers of subjects, Holter sessions, and 30-min intervals successfully evaluated for ST-segment depression and HRV. One control participant had no successful electrocardiogram session because she removed the Holter monitor and subsequently dropped out of the study. We removed 82 extreme measures out of 11,218 because of excessive tachycardia, premature ventricular depolarization, or artifacts due to interference and lead loss. Although we were able to recruit more control than intervention subjects, we obtained similar numbers of Holter sessions in each group during the trial period (July 2003 through December 2004), because of the design feature of measuring one control and one intervention subject on each study day. Among 55 (79%) of the control participants, we took additional measures after they received stoves at the end of the trial.

**Table 2:** Electrocardiogram measures for between-groups comparisons during the trial period and before-and-after comparisons among the control group.

Between-groups			Before-and-after		
Parameter	Control (open fire)	Intervention (chimney)	Before (open fire)	After (chimney)
Session measures (*n*)								
Subjects		70		49		55		55
20-hr sessions		110		112		88		65
30-min measures		4,256		4,333		3,429		2,547
ST measures								
ST value [mm (mean ± SD)]		–0.04 ± 0.62		0.06 ± 0.54		–0.08 ± 0.63		–0.04 ± 0.52
ST depression events (*n*)*a*		272		105		243		80
ST depression rate [events per person-day (*n*)]		3.1		1.2		3.4		1.5
Heart rate [beats/min (mean ± SD)]								
Overall		73 ± 12		72 ± 13		73 ± 13		72 ± 12
During ST depression		77 ± 15		83 ± 19		78 ± 15		82 ± 15
HRV [GM (GSD)]								
SDNN (msec)		82.2 (1.5)		82.2 (1.6)		82.2 (1.5)		83.3 (1.5)
HF (msec^2^)		453 (3.6)		412 (4.2)		450 (3.6)		526 (3.4)
LF (msec^2^)		705 (3.9)		660 (4.4)		709 (3.8)		765 (4.0)
Abbreviations: GM, geometric mean; GSD, geometric SD. **a**Events defined as 30-min average ST-values < –1.00 mm, regardless of slope.								

We evaluated the ST-segments for transient horizontal or down-sloping depression, consistent with myocardial ischemia, but only 36 of these events occurred, and most were among only three subjects. There were too few of these events to warrant statistical analyses, so the models we present focus on the nonspecific outcome of 30-min average ST-segments below –1.0 mm, regardless of slope.

The mean ST-segment values were approximately 0.10 mm lower, and there were almost three times more 30-min periods with average ST-segment values ≤ –1.0 mm, among the control group than among the intervention group (Table 2). These nonspecific ST-depression events occurred among 19 people in the control group and 19 people in the intervention group. Among the 55 control subjects in the before-and-after study, the number of people experiencing ST-segment depression events did not change between study periods; however, the mean ST-segment level was higher and the rate of depression events per person-day was reduced to less han half after they received the chimney stove. We observed no clear or consistent difference in HRV measures between groups or study periods.

Table 3 presents the results from logistic models for ST-segment depression. In the between-groups study, the randomized chimney-stove intervention was associated with large reductions in the odds of ST-segment depression in both crude and adjusted models. In the before-and-after study, we found an association between ST-segment depression and chimney stove remarkably similar to the between-groups result, particularly after adjustment for potential time-varying confounders.

**Table 3:** Odds ratios (ORs) for nonspecific ST-segment depression (30-min average ≤ –1 mm, regardless of slope) associated with chimney-stove intervention compared with open fire from two study designs: between-groups and before-and-after analyses.

Crude			Adjusted		
Comparison	OR (95% CI)	*p*-Value	OR (95% CI)	*p*-Value
Between-groups		0.34 (0.15, 0.81)		0.015		0.26 (0.08, 0.90)*a*		0.033
Before-and-after (only control group)		0.41 (0.24, 0.70)		0.001		0.28 (0.12, 0.63)*b*		0.002
**a**Adjusted for age (quadratic), BMI (quadratic), asset index category, ever smoking, SHS, owning a wood-fired sauna, recent use of wood-fired sauna, and time of day (natural spline with 5 degrees of freedom). **b**Adjusted for age (quadratic), day of week, season (wet/dry), daily average temperature and relative humidity, daily rainfall, interactions of weather variables with season, recent use of wood-fired sauna, and time of day (natural spline with 5 degrees of freedom).								

**Figure 3:**
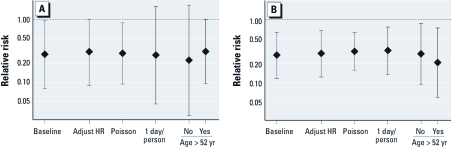
ORs (95% CI) for nonspecific ST-segment depression (30-min average ≤ –1.0 mm, regardless of slope) associated with chimney-stove intervention from sensitivity analyses of between-groups (*A*) and before-and-after (*B*) comparisons. Baseline models are the adjusted results from Table 3. Sensitivity analyses, from left to right: adjustment for 30-min average heart rate (HR); use of Poisson model for the counts of 30-min intervals with ST values ≤ –1.0 mm per session; inclusion of only the last electrocardiogram session per subject-period; and estimation by age categories.

In both the between-groups and before-and-after study designs, we found similar results after either adjusting for heart rate, using a Poisson model for the rate of ST-segment depression, or including only one Holter session per subject (Figure 3). The odds ratios (ORs) for ST-segment depression associated with the stove intervention were similar among the age groups (< 52 and ≥ 52 years) and BMI groups (< 25 and ≥ 25 kg/m^3^) in both study designs. For the between-groups comparison, we found that the association between stove and ST-segment depression was significantly greater after the first 3 months of follow-up (*p*-value for interaction = 0.034), when the odds of ST-segment depression in the chimney-stove intervention group was 0.16 times lower than in the control group (95% CI: 0.04, 0.61).

Table 4 shows the estimated percent differences in HRV associated with the chimney-stove intervention. We found no evidence of an association between HRV and the stove intervention based on between-groups comparisons. Using the before-and-after design, HF was increased when the chimney stove was used based on the unadjusted model (21.6 msec; 95% CI: –0.2, 48.3; *p* = 0.053), but the association was weaker after adjustment for confounders (13.9 msec; 95% CI: –10.2, 44.5). The before-and-after comparison did not change appreciably when we restricted it to each subject’s first Holter session per study period, and we found no evidence of an interaction with age group or BMI (data not shown).

**Table 4:** Difference in HRV associated with the chimney-stove intervention compared with open fire from two study designs: between-groups and before-and-after.

Crude			Adjusted		
Comparison and HRV measure	Mean (95% CI)	*p*-Value	Mean (95% CI)	*p*-Value
Between-groups								
SDNN difference (msec)		–0.1 (–8.5, 9.0)		0.979		2.0 (–6.5, 11.2)*a*		0.660
HF difference (msec^2^)		–8.9 (–32.9, 23.7)		0.552		–2.4 (–25.5, 27.9)*a*		0.860
LF difference (msec^2^)		–6.4 (–30.8, 26.5)		0.667		1.7 (–23.2, 34.7)*a*		0.905
Before-and-after (only control group)								
SDNN difference (msec)		2.1 (–3.6, 8.1)		0.476		0.9 (–6.0, 8.3)*b*		
HF difference (msec^2^)		21.6 (–0.2, 48.3)		0.053		13.9 (–10.2, 44.5)*b*		
LF difference (msec^2^)		12.9 (–6.6, 36.4)		0.211		7.7 (–14.3, 35.4)*b*		0.525
**a**Adjusted for age (quadratic), BMI (quadratic), asset index category, ever smoking, SHS, owning a wood-fired sauna, recent use of wood-fired sauna, heart rate, and time of day (natural spline with 5 degrees of freedom). **b**Adjusted for age (quadratic), day of week, season (wet/dry), daily average temperature and relative humidity, daily rainfall, interactions of weather variables with season, heart rate, recent use of wood-fired sauna, and time of day (natural spline with 5 degrees of freedom).								

## Discussion

To the best of our knowledge, RESPIRE was the first randomized controlled trial of an intervention to reduce air pollution exposures in a general population under normal conditions (Smith et al. 2006, 2010). Reductions in indoor biomass smoke were maintained over a period of many months by introduction of chimney stoves in households previously cooking over open fires. We recruited women from the intervention and control households and examined whether the chimney-stove intervention was associated with occurrence of ST-segment depression or measures of HRV. After adjusting for several potential confounders, we found that the rate of nonspecific ST-segment depression was 74% lower among women from intervention households during the trial period. Using a complementary study design examining this association within subjects, we similarly observed that women had a 72% lower rate of ST-segment depression events after receiving the intervention stove. We found no significant association between the stove intervention and HRV. Given the fundamental differences between these two study designs in the potential sources of bias, the findings together strongly suggest that the wood smoke exposure-reduction intervention reduced the occurrence of ST-segment depression.

To our knowledge, this is the first study to investigate associations between electrocardiogram measurements and wood smoke or any long-term (several months) air pollution exposure. Our findings add to the growing evidence that pollutants from combustion of biomass produce many health impacts similar to those from fossil fuels and tobacco smoke (Naeher et al. 2007). The public health implication is grave because these other forms of combustion-generated air pollution have been linked to cardiovascular morbidity and mortality in numerous studies (Brook et al. 2004; Pope et al. 2009; Tonne et al. 2007; U.S. Department of Health and Human Services 2006) and because high exposures to indoor air pollution from household use of solid biomass fuels are common in poor countries.

Associations between daily fluctuations in ambient particles and electrocardiogram changes related to ventricular repolarization, such as ST-segment depression, have been observed in previous studies. Among 45 subjects with stable coronary heart disease in the Helsinki ULTRA study, risk of ST-segment depression during exercise tests was associated with particulate air pollution 2 days before the clinic visit (Pekkanen et al. 2002), and the association was stronger with PM_2.5_ originating from local combustion sources (Lanki et al. 2006). Among active elderly Bostonians, each observed up to 12 times during a 4-month period, ambient black carbon concentration during the previous 12 hr and 5 hr before testing was associated with increased risk of ST-segment depression among subjects with at least one ST-depression episode (Gold et al. 2005). In the present study, we examined the association between stove intervention and ST-segment depression among the subset of 38 control and intervention subjects with at least one 30-min average ST value < –1.0 mm and found a stronger association (OR = 0.17 vs. 0.26; data not shown), similar to findings by Gold et al. (2005) on the association with ambient black carbon. In a double-blind, randomized crossover study among 20 men with prior myocardial infarction, Mills et al. (2007) found that 1-hr diesel exhaust exposure during exercise was associated with greater ischemic burden, as measured by the magnitude and duration of ST-segment depression. Henneberger et al. (2005) found that ambient particle levels were associated with changes in repolarization duration, morphology, and variability, which could produce the type of ST-segment changes that we observed.

The mechanisms by which combustion-generated particles affects cardiovascular function are not entirely understood, but oxidative stress and inflammation are central in leading hypotheses (Brook et al. 2004; Chuang et al. 2007). Although the toxicological evidence on wood smoke is less complete than that on ambient particles, experimental evidence suggests that wood smoke influences similar pathways. Using a controlled experiment among healthy adults, Barregard et al. (2006) found increased systemic inflammation markers, blood coagulation factors, and lipid peroxidation after 4-hr wood smoke PM_2.5_ exposures in the range of 240–280 μg/m^3^, levels similar to the daily averages we observed among women cooking over open fires. Lai and Kou (1998) found that wood smoke stimulates bronchopulmonary C-fibers, which have been shown to be involved in the pulmonary reflexes that in rats mediate both cardiac oxidative stress and electrophysiological changes due to ambient particles (Ghelfi et al. 2008). Therefore, it is plausible that wood-smoke–induced stimulation of pulmonary reflexes leading to oxidative stress may have contributed to the electrocardiographic changes we observed. Specifically, elevated oxidative stress resulting in alterations in myocardial action potentials and changes in ventricular repolarization may explain the greater frequent ST-segment depression among women cooking over open fires. Future studies should examine whether air-pollution–induced changes in ST-segment values and other electrocardiogram measures, such as QT intervals and T-wave morphology, occur in association with increases in cardiac oxidative stress.

Although previous evidence of associations between air pollution and HRV motivated our study (Chahine et al. 2007; Chuang et al. 2007; Pope et al. 1999; Schwartz et al. 2005), this is not the first study to fail to find evidence of overall association between air pollution and HRV. Wheeler et al. (2006)found that ambient particles in Atlanta, Georgia, were associated with increased HRV among individuals with chronic obstructive pulmonary disease, and with decreased HRV among individuals with recent myocardial infarction. Other studies have reported that air pollution–HRV associations were significantly modified by genetic or environmental factors (Park et al. 2006, 2008). Therefore, our null findings may be explained by the participants not being susceptible to the autonomic effects of combustion particles, perhaps because they were generally healthy, or by opposing associations within subgroups of the study population. We did not find any consistent evidence of effect modification by age or BMI on the association between stove intervention and HRV (data not shown). Another potential explanation for the lack of evidence of an association between the stove intervention and HRV is that wood smoke particles may have only short-term effects on HRV without changing a person’s average HRV level. A final possibility is that there may be an exposure level above which reductions in PM_2.5_ will not substantively improve HRV, and the average PM_2.5_ exposure in the intervention group (102 μg/m^3^), which was much higher than ambient levels associated with HRV in previous studies (Chahine et al. 2007; Chuang et al. 2007; Pope et al. 1999; Schwartz et al. 2005; Wheeler et al. 2006), may be within this plateau region in the exposure–response association.

A key feature of our study, in contrast to the above ambient air pollution studies that evaluated short-term associations, is that we examined differences in electrocardiograph measures between groups or periods with persistently high or low exposure levels. We aimed to address a relevant policy question about the effect of a more permanent change in air pollution exposure. With the between-groups study design, the exposure concentration contrast between groups was substantial, roughly a mean difference of 164 μg/m^3^ PM_2.5_, and was created by a stove intervention that we assigned randomly, reducing the likelihood of confounding. Consistent with the smaller contrast in exposure we observed during the first 3 months of the trial compared with the rest of the trial, we found that the effect of the chimney stove on ST-segment depression was significantly greater after 3 months of follow-up, suggesting that the full benefit of the stove may be achieved after an adaptation period. With the before-and-after study design, a mean within-subject reduction in PM_2.5_ exposure concentration of 92 μg/m^3^ was attained and subjects served as their own controls, eliminating concern of confounding by between-subject differences.

Although the two study designs we employed have major strengths, they are not without potential limitations. We recruited the women from randomized groups, but the number of subjects was not large, and the proportion of eligible women participating was differential by study group (75% for control group, 54% for intervention group). If participation was also associated with ST-segment depression or HRV, selection bias may have produced study groups that were not comparable. We found, however, that the study groups were similar according to all baseline covariates we analyzed (Table 1). Moreover, as reported previously, we found no meaningful difference between the baseline characteristics of eligible women in the RESPIRE trial according to whether they participated in this cardiovascular add-on study (McCracken et al. 2007). The before-and-after comparisons rely on the assumption that, conditional on time-varying covariates included in the models, ST-segment and HRV values would not have changed in the control subjects over time if the chimney-stove intervention had not occurred. We feel that this is a reasonable assumption, but unmeasured time-varying confounders cannot be ruled out.

The exposure assessment strategy we employed was successful for estimating the average effect of the stove intervention on personal PM_2.5_, but there are two important limitations that should be addressed in future studies. The first is that the few personal exposure measures we took do not allow accurate estimation of each subject’s typical exposure during the trial or of their time-resolved exposures over several days before electrocardiogram sessions. Based on analyses of child exposures in RESPIRE, which had similar between- and within-subjects variance components (data not shown), McCracken et al. (2009) showed that, because of wide daily variability in exposure, a larger number of repeated measures would be required to provide estimates of a subject’s long-term (over several months) exposure that are accurate enough for use in exposure– response modeling of chronic health effects. In addition, assessment of recent exposure history, perhaps hourly for up to 5 days before each Holter session, would allow analyses that are more sensitive for detection of acute health effects that may occur over several hours or days. The second limitation of our exposure assessment strategy is that we did not measure potentially important pollutants, such as ultrafine particles, the volatile aldehydes and ketones in wood smoke, or particle-bound polyaromatic hydrocarbons. It would be valuable to characterize the specific wood smoke constituents that have cardiovascular effects because some interventions may reduce some constituents more than others, particularly when the combustion conditions or fuels are substantially modified.

The clinical significance of 30-min average ST-segment values below –1 mm in this population is unclear. The classical definition of ST-segment depression indicative of overt ischemia requires an episode that is reversible and ST-segments that are horizontal or downward sloping, usually assessed during exercise stress testing (Pálinkás et al. 2002). However, Cannon et al. (1997) evaluated hospital admission electrocardiogram recordings among resting subjects and found that ST-segment values ≤ –1 mm and ≤ –0.5 mm predicted death or myocardial infarction within 1 year, without taking the slope of the ST segment into account. Moreover, although we did not incorporate an exercise protocol, subjects in our study live in a hilly area at high altitude and are involved in agricultural work, firewood collection, and washing of clothes by hand, among other strenuous activities that are likely to challenge that cardiovascular system. Nevertheless, we conclude that the differences in the rates of ST-segment depression we observed represent a change in ventricular repolarization but acknowledge that the potential pathophysiology underlying these changes is unknown and may or may not involve ischemia. We cannot rule out the possibility that these differences may have been caused by subject position (e.g., open fires are typically on the floor, and the improved stove is used while standing) or other technical factors not related to air pollution, but studies of ambient particle exposures at much lower concentrations, including one study using the same type of ST-segment depression measure among people conducting their everyday activities (Chuang et al. 2008) and another study finding nonspecific ST changes among healthy subjects at rest (Zhang et al. 2009), support the conclusion that fine combustion particles in wood smoke are responsible for the associations we observed with the improved stove intervention. Controlled exposure studies with more detailed clinical evaluations may prove useful in clarifying the etiology and prognostic value of ST-segment depression associated with biomass smoke exposure.

To our knowledge, the RESPIRE trial was the first study to examine the association between exposure to wood smoke from household stoves and an electrocardiographic outcome. The risk of ST-segment depression associated with use of traditional open fires may have health implications for the nearly 3 billion people living in rural areas of less developed countries who rely on solid biomass fuels. Household air pollution in developing countries is already estimated to be a leading risk factor for disease globally, because of evidence of respiratory effects (Smith et al. 2004), and our study adds to concern that previous risk assessments may have underestimated this burden by not including cardiovascular health effects.
